# Allogeneic hematopoietic stem cell transplantation for pediatric acute myeloid leukemia in first complete remission: a meta-analysis

**DOI:** 10.1007/s00277-022-04965-x

**Published:** 2022-08-30

**Authors:** Riccardo Masetti, Edoardo Muratore, Davide Gori, Arcangelo Prete, Franco Locatelli

**Affiliations:** 1 Pediatric Oncology and Hematology Unit “Lalla Seràgnoli, IRCCS Azienda Ospedaliero-Universitaria Di Bologna, Bologna, Italy; 2 Department of Biomedical and Neuromotor Sciences (DIBINEM), University of Bologna, Bologna, Italy; 3 Department of Pediatric Hematology/Oncology and Cell and Gene Therapy, IRCCS Ospedale Pediatric Bambino Gesù, Sapienza University of Rome, Rome, Italy

**Keywords:** Acute myeloid leukemia, Allogeneic hematopoietic stem cell transplantation, Pediatrics

## Abstract

Identification of pediatric patients with acute myeloid leukemia (AML) candidates to receive allogeneic hematopoietic stem cell transplantation (allo-HSCT) in first complete remission (CR1) is still a matter of debate. Currently, transplantation is reserved to patients considered at high risk of relapse based on cytogenetics, molecular biology, and minimal residual disease (MRD) assessment. However, no randomized clinical trial exists in the literature comparing transplantation with other types of consolidation therapy. Here, we provide an up-to-date meta-analysis of studies comparing allo-HSCT in CR1 with chemotherapy alone as a post-remission treatment in high-risk pediatric AML. The literature search strategy identified 10 cohorts from 9 studies performing as-treated analysis. The quantitative synthesis showed improved overall survival (OS) (relative risk, 1.15; 95% confidence interval [CI], 1.06–1.24; *P* = 0.0006) and disease-free survival (relative risk, 1.31; 95% CI, 1.17–1.47; *P* = 0.0001) in the allo-HSCT group, with increased relapse rate in the chemotherapy group (relative risk, 1.26; 95% CI, 1.07–1.49; *P* = 0.006). Sensitivity analysis including prospective studies alone and excluding studies that reported the comparison only on intermediate-risk patients confirmed the benefit of allo-HSCT on OS. Further research should focus on individualizing allo-HSCT indications based on molecular stratification and MRD monitoring.

**Supplementary Information:**

The online version contains supplementary material available at 10.1007/s00277-022-04965-x.

## Introduction

Acute myeloid leukemia (AML) is the second most common leukemia in children, accounting for approximately 20% of pediatric leukemias [1, 2]. Treatment outcomes have improved significantly over the past 30 years, with current survival rates approaching 70% [3, 4].

This achievement is mainly due to the cooperative efforts of multiple international study groups that have led to a significant improvement in patient stratifications with a subsequent optimal refinement of the risk-adapted therapy, and thanks to the refinements in supportive care [3, 5]. Nowadays, up to 85–90% of children with AML achieve a first complete remission (CR1) with a standard induction chemotherapy approach [6]. However, the cumulative incidence of relapse is still high, ranging between 25% and 35% in the major collaborative groups protocols [3], underlying the need for further improvement in the post-induction consolidation treatment.

Allogeneic hematopoietic stem cell transplantation (allo-HSCT) has been widely used as post-remission therapy [7]. In adults, allo-HSCT significantly improves relapse-free survival and overall survival (OS) in intermediate- and poor-risk AML in CR1 compared to chemotherapy alone [8]. In children, the benefit of allo-HSCT as a consolidation strategy for patients with newly diagnosed AML remains controversial [9]. Deciding the optimal indication for allo-HSCT in children is a delicate balance between the risk of relapse, the non-relapse mortality risk, and the wide plethora of late effects related to the procedure [5]. Currently, a consensus of which children would benefit of allo-HSCT in CR1 is lacking, partly because no randomized clinical trial exists in the literature comparing transplantation with other types of post-remission therapy.

In the 1990s, many protocols applied a Mendelian/genetic randomization, reserving allo-HSCT in CR1 to those children who had an available matched sibling donor (MSD). A meta-analysis of these studies published in 2002 found that patients allocated to allo-HSCT on the basis of having an MSD available had a reduced relapse risk (RR) and an improved disease-free survival (DFS) and OS [10]. However, subsequent studies challenged this idea, finding better DFS but no difference in OS for patients transplanted in CR1 compared to chemotherapy alone [5, 11]. A systematic review published in 2010 by Niewerth et al. summarized phase 3 clinical trials using intention-to-treat analysis or as-treated analysis adjusted for time to transplantation, with the majority of the studies included applying the Mendelian/genetic randomization. Allo-HSCT resulted in significantly lower RR but higher transplant-related mortality (TRM) and more long-term toxicity than chemotherapy alone as consolidation therapy. OS was therefore comparable, and the authors calculated that 10 patients should receive allo-HSCT in order to avoid one relapse [6].

Based partly on these results and mainly on a better risk-based stratification of subgroups, contemporary protocols have removed the availability of MSD as an indication for allo-HSCT in CR1 for all patients [4, 6, 12]. Moreover, the results of allo-HSCT from matched unrelated donors (MUD) have progressively improved and are now considered superimposable to the ones obtained using an MSD as donor [13, 14]. With the aim of reserving transplantation for the subset of patients with high relapse risk, disease assessment based upon disease characteristics and response-related factors has been progressively implemented. The analysis of the combined data from the clinical trials POG 8821, CCG 2891 and 2961, and MRC 10 revealed that allo-HSCT from MSD improved DFS and OS only in intermediate-risk AML. However, the small number of patients included in the high-risk group precluded any definitive conclusions from being drawn regarding this population [15].

Nowadays, the improvement in the understanding of the genetic basis of AML has led to an enhanced implementation of molecular biology and cytogenetics into risk stratification [3, 4, 16]. Moreover, minimal residual disease (MRD) measurement by either multiparametric flow cytometry or molecular biology may help identify patients at higher risk of relapse and benefitting from allo-HSCT in CR1 [17].

There is general agreement that allo-HSCT must not be recommended in CR1 for children with Down syndrome, acute promyelocytic leukemia, and core-binding factor leukemia, namely AML with t(8;21), inv(16), and t(16;16) [6]. For the remaining subgroups of patients, the international groups’ definition of intermediate and high risk differs, together with allo-HSCT indications. Moreover, not enough data are available in the present literature to clearly define the impact of allo-HSCT in CR1 on outcomes in patients considered at intermediate and high risk of relapse. Therefore, deconvoluting the benefit of allo-HSCT in newly diagnosed leukemia with higher relapse risk remains a challenge, especially taking into account the novel biological categories uncovered by next-generation sequencing technologies, of which the prognostic impact still needs to be validated in prospective pediatric cohorts [16, 18, 19, 20].

The aim of this paper is to provide a meta-analysis of prospective and retrospective studies comparing allo-HSCT vs chemotherapy alone for children with AML in CR1 at higher risk of relapse.

## Methods

### Literature search

The present meta-analysis was conducted according to the Preferred Reporting Items for Systematic Reviews and Meta-Analyses (PRISMA) guidelines [21].

Electronic databases, namely PubMed and Trip, were searched the 11/09/2021 in order to identify relevant studies. The string used to perform the search is provided in the Supplementary Material.

The search was restricted to English language studies involving AML pediatric patients addressing the use of allo-HSCT in CR1. We included both prospective and retrospective studies that were performed an as-treated analysis.

Two reviewers (E.M. and R.M.) independently identified potentially eligible studies by screening titles and abstracts. The same authors assessed the full texts of potentially relevant studies for inclusion and consulted the references of previously published primary and secondary papers to manually search for additional relevant papers. Any disagreement regarding eligibility and inclusion in the systematic review was resolved through discussion and consensus between the 2 readers. If consensus was not reached, the opinion of a third author (D.G.) who acted as a “blind” final arbiter was requested. Investigators and corresponding authors were contacted to obtain additional information about studies with incomplete data.

### Data extraction and meta-analysis

We used the same methodology for data extraction, performed independently by the same 2 reviewers (E.M. and R.M.) with the help of the third author (D.G.) if disagreement occurred. Data were summed and analyzed using Microsoft Office Excel 2013 (Microsoft, Redmond, WA). Subsequently, we performed a meta-analysis of data regarding the comparison between allo-HSCT and other types of consolidation therapy in CR1 for high-risk patients. The different outcomes that were reported consistently enough in the studies to be considered eligible for the meta-analysis and were, therefore, included in the quantitative synthesis are OS, RR, and DFS. If multiple articles reported results from the same cohort, the most recent data were analyzed. Studies including young adults and not reporting separately their outcome were not excluded from the analysis.

We analyzed statistical heterogeneity to determine the feasibility of summing the results of the different included studies. We assessed heterogeneity by graphic funnel plots and by calculating the I2 statistic. An I2 statistic > 50% was considered significantly heterogeneous. When the number of studies was < 5 or studies were substantially heterogeneous, we used a random-effects model in accordance with the Cochrane Handbook for Systematic Reviews of Interventions [22, 23]. We followed the method of DerSimonian and Laird [22, 23] to compute the random-effects estimates for the corresponding statistics. We chose to use forest plots to graphically show effect estimates with 95% confidence intervals for individual trials and pooled results. We carried out the meta-analysis using RevMan version 5.3 (https://revman.cochrane.org).

### Quality assessment

We used the Strengthening the Reporting of Observational Studies in Epidemiology (STROBE) statement to assess the study quality of the experimental and observational original studies included in this meta-analysis. We opted not to use the Cochrane Tool for Quality Assessment because no inserted randomized study performed the randomization on the use of allo-HSCT. The STROBE statement is a 22-item tool designed to evaluate the quality of observational studies [24]. Items are associated with different sections of an article, such as title and abstract (item 1), introduction (items 2 and 3), methods (items 4–12), results (items 13–17), discussion (items 18–21), and other informations (item 22 for funding). Eighteen items are identical for 3 different study designs, whereas 4 items (items 6, 12, 14, and 15) are differentially intended for a specific study type (i.e., cohort or case–control study). The STROBE statement does not provide scoring stratification itself, but the higher the score, the higher the quality of the study is considered. As previously described [25], we therefore utilized 3 score thresholds, corresponding to 3 levels of quality: 0 to 14 was considered poor quality; 15 to 25, intermediate quality; 26 to 33, good quality.

## Results

### Literature search

The literature search strategy identified 2141 references (1143 in PubMed, 997 in Trip and one identified through manual search).

**Fig. 1:**
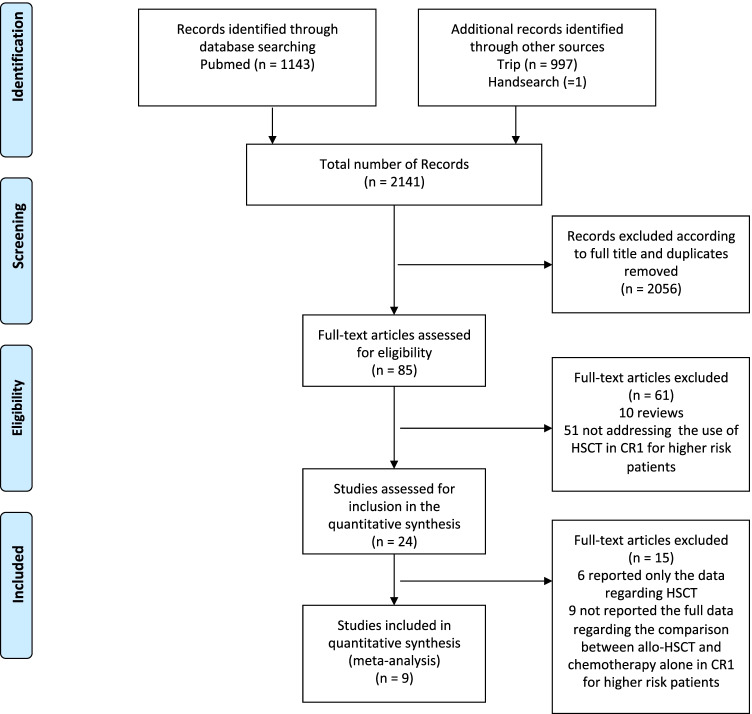
PRISMA flow diagram of the search strategy and included studies. The relevant number of papers at each point is given

Potentially relevant papers were identified by full titles and abstracts. Full-text articles assessed for eligibility were 85 (Fig. 1). Among these 85 studies, 10 were excluded because they were reviews, and 51 because they did not address the use of allo-HSCT in CR1 for higher-risk patients. Therefore, 24 studies were further assessed for inclusion in the quantitative synthesis. We selected trials that did not apply only a Mendelian/genetic randomization as allo-HSCT indication in CR1, but otherwise compared transplantation from any donor and chemotherapy in high-risk patients based on cytogenetics, molecular biology, and MRD (Supplementary Table 1). Considering that risk definition and allo-HSCT indications varied between different protocols, in the present study, high-risk patients were considered the one allocated to receive allo-HSCT based on each trial risk assessment.

Included papers reported prospective and retrospective studies assigning pediatric patients with AML in CR1 to undergo allo-HSCT vs non-allo-HSCT treatment, using as-treated analysis to compare outcomes (Table 1). To perform the meta-analysis, we excluded from the eligible studies 15 papers: 6 reported only the data regarding allo-HSCT, while 9 did not report the full data regarding the comparison between the two groups.

**Table 1:** Summary of included studies. Quality assessed using the Strengthening the Reporting of Observational Studies in Epidemiology (STROBE) for prospective cohorts

Study group	Study protocol	Reference	Enrollment period	Study design	Analysis	allo-HSCT group, *n*	Chemotherapy group, *n*	Quality assessment
Japan	AML 99	Tsukimoto et al. 2009	2000–2002	Prospective	As treated	37	69	Good
SJCRH	AML 02	Rubnitz et al. 2010	2002–2008	Prospective	As treated and adjusted for time to transplantation	48	31	Good
BFM	AML 1998	Klusmann et al. 2012	1998–2003	Prospective	As treated and adjusted for time to transplantation	60	172	Good
AIEOP	AML 2002/01	Pession et al. 2013	2002–2011	Prospective	As treated and adjusted for time to transplantation	141	139	Good
COG	POG 9421, CCG 2961,AAML03P1	Kelly et al. 2014	1998–2006	Retrospective	As treated and adjusted for time to transplantation	54	77	Intermediate
COG	AAML0531	Gamis et al. 2014	2006–2010	Prospective	As treated	93	382	Good
Japan	AML 05	Hyakuna et al.2019	2006–2010	Retrospective	As treated	45	6	Intermediate
Uruguay	LAM 08	Alvarez et al. 2020	2008–2017	Retrospective	As treated	10	22	Low
South Korea	Different protocols	Lee et al. 2021	2000–2013	Retrospective	As treated	34	28	Low

In the 9 studies in which quantitative analyses were performed, the only outcomes reported consistently enough to allow for a meta-analysis were OS, RR, and DFS.

Two groups were compared: the allo-HSCT group received transplantation from any donor as consolidation therapy, and the chemotherapy group received additional chemotherapy cycles and/or auto-HSCT (Supplementary Table 1).

Five articles reported the results of prospective trials [26–30], while the remaining 4 were retrospective analyses [31–34]. In the case of two papers reporting results from different trials and different time spans [32, 34], we selected the data corresponding to our inclusion criteria which were reported separately. Klusmann et al. described the results of a trial applying Mendelian/genetic randomization [27], but they reported separately the data regarding high-risk patients transplanted from any donor, and we selected to include such data (Supplementary Table 1). The study performed by Gamis et al. included patients who did or did not receive gemtuzumab ozogamicin [30], and we opted to include the data inserted in this paper as two different cohorts.

The quality of the included clinical studies was assessed as described in “Methods”. All of the 5 prospective studies were rated as good quality. Of the 4 retrospective studies, 2 were rated as intermediate quality and 2 as low quality (Table 1).

### Overall survival

**Fig. 2:**
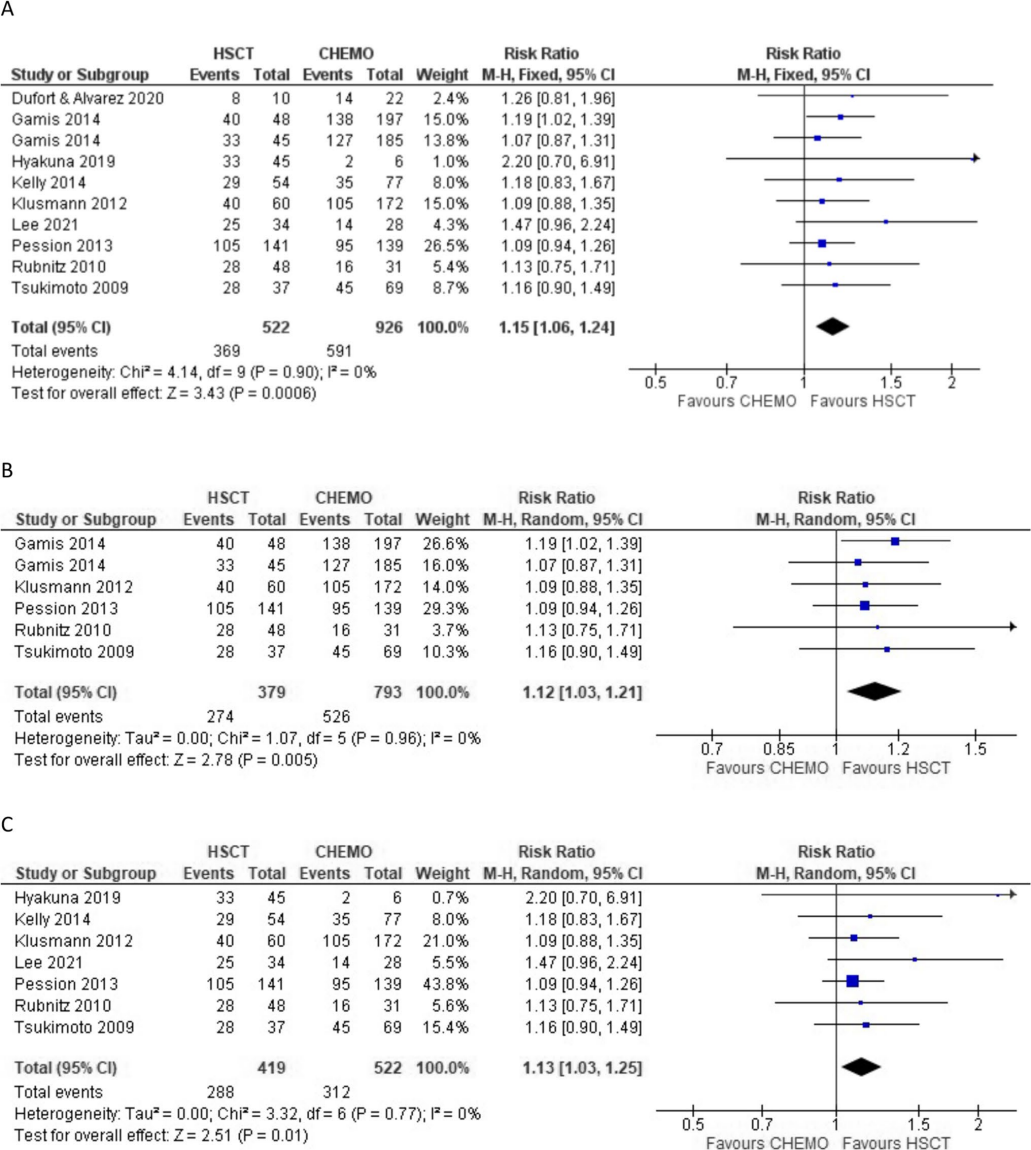
Forest plot showing the association between allo-HSCT and improved OS in higher-risk pediatric AML in CR1. (**A**) All included studies. (**B**) Only prospective trials. (**C**) Excluding studies including only IR patients. HSCT, hematopoietic stem cell transplantation

All studies included in the meta-analysis reported OS endpoints with a total of 1448 patients, 522 and 926 in the allo-HSCT and chemotherapy group, respectively. Patients allocated in the allo-HSCT group presented significantly improved OS, with a relative risk of 1.15 (95% confidence interval [CI], 1.06–1.24; *P* = 0.0006). Heterogeneity among studies was absent (0%) (Fig. 2A).

We further evaluated the included studies with sensitivity analysis. We performed the quantitative synthesis selecting only the prospective trials, as previously done in adult patients [8]. We included 6 different cohorts from 5 studies [26–30] all rated as good quality of evidence according to the STROBE statement; 379 and 793 patients were allocated in the allo-HSCT and chemotherapy group respectively. Allo-HSCT was still associated with improved OS, as the pooled results showed a relative risk of 1.12 (95% CI, 1.03–1.21; *P* = 0.005) with 0% heterogeneity (Fig. 2B). We then excluded the two studies that reported the comparison only in intermediate-risk patients [30, 34], therefore including the papers that analyzed high-risk AML only. In this subgroup analysis that included 941 patients, improved OS in the allo-HSCT group was still observed with a relative risk of 1.13 (95% CI, 1.03–1.25; *P* = 0.01) and absent heterogeneity (Fig. 2C).

### Relapse rate

We analyzed the impact of allo-HSCT on RR, performing quantitative synthesis including studies that reported RR among the outcomes.

**Fig. 3:**
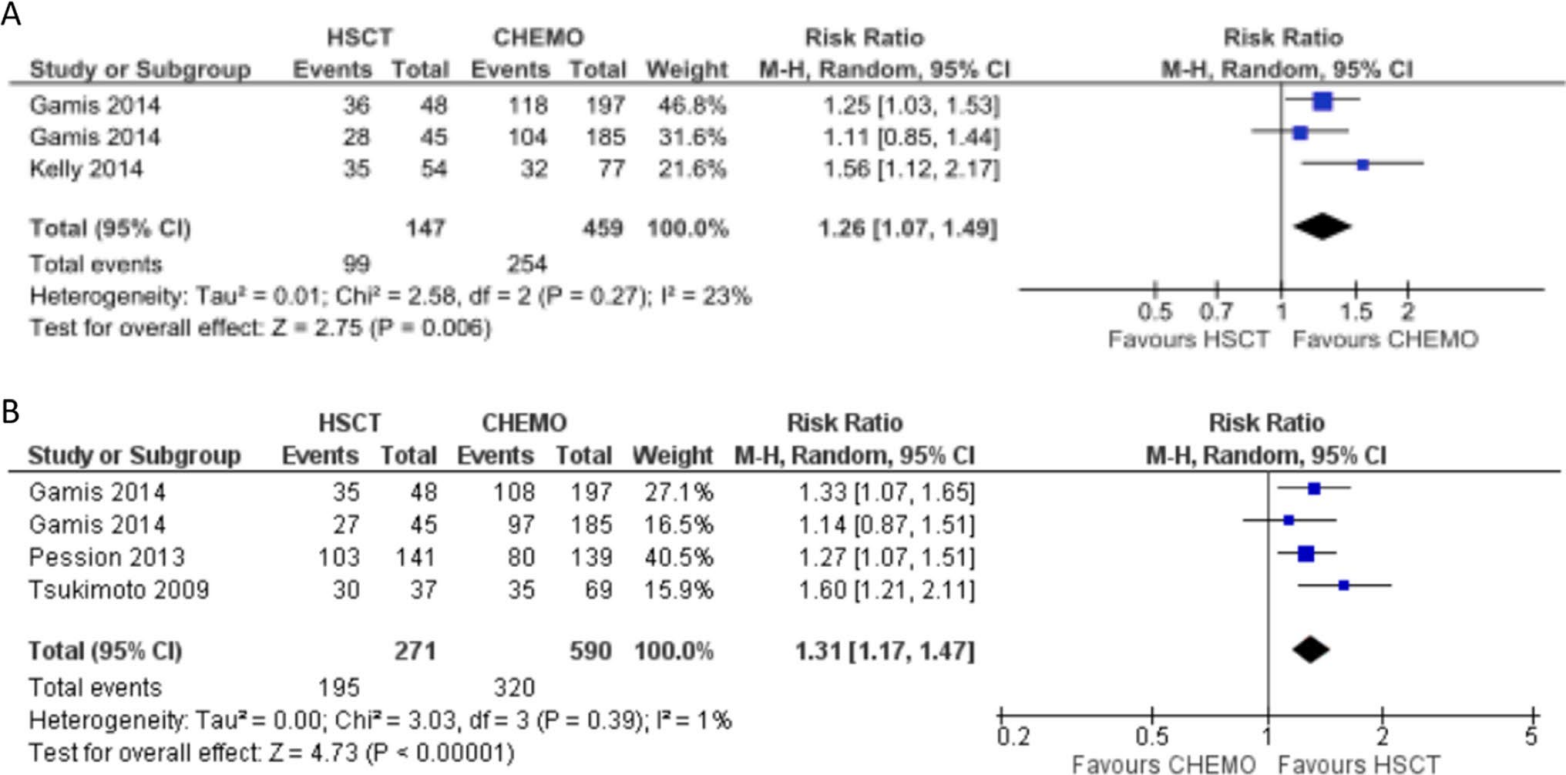
Forest plot showing the association between allo-HSCT and reduced relapse-related outcomes in higher-risk pediatric AML in CR1. (**A**) RR, (**B**) DFS. DFS, disease-free survival; HSCT, hematopoietic stem cell transplantation; RR, relapse rate

Three different cohorts from 2 of the 9 studies included in the meta-analysis reported the RR [30, 32]. The number of patients for this analysis was 147 and 459 in the allo-HSCT and chemotherapy group, respectively, for a total of 606 patients. The RR was significantly higher in the chemotherapy group than in the allo-HSCT group, with a relative risk of 1.26 (95% CI, 1.07–1.49; *P* = 0.006). Heterogeneity among the cohorts was 23% (Fig. 3A).

### Disease-free survival

We analyzed the impact of allo-HSCT on DFS, performing quantitative synthesis including studies that calculated DFS of the two groups considered in the present meta-analysis.

DFS was reported in 3 out of the 9 included studies, for a total of four cohorts and 861 patients. Of them, 271 were allocated to the allo-HSCT group and 590 to the chemotherapy group. All the cohorts considered derived from prospective studies rated as good quality of evidence according to the STROBE statement.

The pooled results showed improved DFS in the allo-HSCT group, with a relative risk of 1.31 (95% CI, 1.17–1.47; *P* = 0.0001). Heterogeneity among studies was low (1%) (Fig. 3B).

## Discussion

Pediatric AML is a heterogeneous disease characterized by the presence of numerous, recurrent cytogenetic and molecular abnormalities with significantly different impact on prognosis [16]. Despite multiple studies over the past 3 decades, the role of allo-HSCT in children with AML in CR1 is still controversial and should be regularly reassessed as the field evolves. In particular, improved risk stratification, thanks to the identification of new prognostic markers and the implementation of MRD monitoring, allowed redefining allo-HSCT indications, while advances in supportive care reduced treatment toxicities [3, 4].

In the present meta-analysis, we addressed the use of allo-HSCT as a post-remission therapy for children with newly diagnosed high-risk AML compared to chemotherapy alone. The analyzed studies included 1448 patients from 1998 to 2017 with higher-risk AML allocated to receive or not allo-HSCT from any donor. We observed improved OS and DFS with reduced RR in the allo-HSCT group. The beneficial effect of allo-HSCT on OS was observed even after removing from the quantitative synthesis the retrospective cohorts or the two studies that included only intermediate-risk patients. Few studies reported DFS and RR, and therefore sensitivity analysis was not possible regarding these outcomes. A consensus statement proposed that in adult patients allo-HSCT should be favored if the projected DFS is expected to improve by at least 10% based on individual’s risk assessment [35]. Considering the higher salvage rate and greater burden of allo-HSCT-related late toxicities in children, Hasle et al. suggested that this cutoff should be significantly greater in pediatric patients [5]. In our study, we observed an improvement of 31% in DFS, therefore justifying the use of allo-HSCT in CR1 according to those statements, as also confirmed by the improved OS found in the allo-HSCT group.

These results are different from the one of a previous systematic review, which observed lower relapse risk but no improvement in OS for patients given allo-HSCT [6]. This may be due to improved transplant-related mortality recorded in recent years, or to the fact that the aforementioned study included mainly trials applying a Mendelian/genetic randomization and not reserving allo-HSCT to higher-risk patients based on risk assessment.

The present results support the use of allo-HSCT in CR1 in pediatric patients considered at higher relapse risk based on cytogenetics, molecular biology, and MRD monitoring, supporting the notion that transplantation can abolish the detrimental impact imparted by specific molecular lesions and poor response to therapy [36]. However, the comparison between the two groups presents several limitations. Allo-HSCT is complicated by more severe long-term toxicity, and salvage rate after relapse is lower in tranplanted patients [5]. Therefore, longer time of follow up is needed in order to fully address the impact of allo-HSCT on OS. In the included studies, the longer time of follow-up was 8 years, while the shorter 3 years (Supplementary Table 1). Moreover, measurements of the quality of life of survivors could further contribute to elucidate the long-term impact of allo-HSCT, an issue of pivotal importance in pediatric patients.

We performed the quantitative synthesis including data only from as-treated analysis. This permitted the comparison of patients according to the treatment they actually received. However, this approach presents some methodological intrinsic pitfalls. Patients allocated to receive allo-HSCT but who experienced early relapse or early treatment-related death in CR are included in the chemotherapy group with this type of analysis. Therefore, more compromised patients often do not undergo allo-HSCT, leading to a selection bias. In the included studies, the primary endpoint of the trial was not always the comparison between allo-HSCT and chemotherapy in higher-risk patients. Accordingly, the number of patients that should have been allocated to allo-HSCT, but instead received chemotherapy in this distinct subgroup, is not clearly described in all studies. In the trials where it was feasible to be extrapolated, it ranges between 5% and 28%, with most studies settling around 12–13% [26, 27, 28, 31, 34]. Therefore, it is definitely not possible estimating the impact of the aforementioned bias in the present dataset.

Correcting as-treated analysis for time to transplantation could reduce this methodological limitation, enabling the researchers to adjust for the events occurring before transplantation [6]. In the included studies, 4 analyzed the data adjusting for time to transplantation (Table 1). Another solution to this problem could be to perform intention-to-treat analysis. Unfortunately, very few studies found in the literature search that matched our inclusion criteria employed this type of analysis. Furthermore, the limitation of intention-to-treat analysis should be taken into account. Non-compliance to the allocated treatment, in particular if a large proportion of participants cross over to the other treatment, impairs the results of this type of analysis [37].

Another limitation to be taken into account consists in the fact that chemotherapy protocols and consolidation strategies in the chemotherapy groups were different among included studies, as well as risk stratification and conditioning regimen deployed (Supplementary Table 1). The effect of different induction and consolidation protocols goes beyond the scope of this paper; however, no clear difference in OS, DFS, and RR was observed between different trials, suggesting that allo-HSCT could exert an effect on survival independently from the chemotherapy protocols applied. The same deduction could be applied to donor choice and availability, as well as the choice of conditioning regimens and donor typing algorithms. In particular, older trials may have performed suboptimal donor choice compared to current perspective, but this should not have affected the conclusions of the present meta-analysis, because it would have tipped the scales in favor of chemotherapy.

The various risk classification strategies can also be considered quite similar, though not identical. However, no universal agreement on the definition of high-risk patients exists among different cooperative groups [9]. In particular, HR definitions has changed considerably during the time span of included studies, and in most protocols, patients allocated in the HR group would be nowadays included in the IR group. Cytogenetic and molecular risk profiling in pediatric AML, especially with the advent of large-scale sequencing techniques, is continuously and rapidly evolving and can help in the effort of individualizing treatment [18]. It is now possible to further stratify outcomes within a known cytogenetic risk group, as in the case of core-binding factor AML. While normally considered lower-risk AML, several studies provided a detailed genomic landscape of this type of pediatric leukemia, uncovering several additional mutations that could affect prognosis [38, 39, 40]. For example, the detection of a *c-KIT* mutation at diagnosis in t(8;21) patients could make this subgroup at higher risk [41, 42] and possibly benefiting from allo-HSCT in CR1 [43]. On the other hand, *KMT2A* rearrangements have a different impact on prognosis dependent not only upon the presence of additional cytogenetic abnormalities [44], but also upon which fusion partner is present [45]. Therefore, allo-HSCT in CR1 could be taken into consideration for patients with poor prognosis *KMT2A* lesions [18]. However, a third of the *KMT2A* rearrangements are detectable only by FISH or molecular methods and not by conventional cytogenetics, highlighting the need to perform an accurate diagnostic panel at AML diagnosis [46]. Recurrent molecular lesions may have prognostic implications as well, most notably *FLT3* mutations. In general, *FLT3*-ITD AML should be considered for allo-HSCT in CR1, but in the subgroup of patients with low allelic ratio and concomitant *NPM1* mutation who achieve MRD negativity after induction therapy transplant indication remains controversial [47].

Some molecular lesions instead confer favorable prognosis. Isolated *NPM1* mutations and biallelic *CEBPα* aberrations are rarely found in pediatric AML patients compared to adults, but they still seem to be associated with improved outcomes [48, 49], and patients carrying these abnormalities should not be offered allo-HSCT in CR1 [9, 18]. Further studies are needed to clearly understand the indication for allo-HSCT in specific molecular subsets of AML. Particularly, only strong cooperative efforts could help shedding some light on the prognostic impact of rare lesion and the eventual benefit of performing transplantation in CR1. Moreover, considering the effect of each somatic mutation per se may be misleading, and the combined effect of co-occurring alterations should be taken into account in risk stratification algorithm. For example, *FLT3*-ITD and *NPM1* mutations in the absence of *DNMT3A* mutations appear to bear a favorable prognosis, while the co-occurrence of *FLT3*-ITD and *WT1* mutations or *NUP98-NSD1* is associated with worse outcomes [16].

MRD monitoring of treatment response has also been shown to be a powerful and independent predictor of relapse in childhood AML [50] and has been increasingly employed to refine risk stratification and indications for allo-HSCT in CR1 [9]. Flow cytometry and molecular methods can be applied to determine MRD. However, molecular MRD determination is currently possible for about 60% of the patients (*FLT3*-ITD, *NPM1* mutation, *RUNX1-2*,*RUNXT1*, *CBFB-MYH11*; *PML-RARA*) [51]. Flow cytometry assessment of the expression of specific leukemia-associated immunophenotypes is the only method that can be used in virtually almost all patients, and therefore is considered the mainstay to determine MRD status [52]. The increasing impact of MRD monitoring on risk assessment could overcome in some cases the initial genetic-driven risk stratification, enabling to re-stratify patients according to therapy response. For example, a child considered at standard risk based on cytogenetics and molecular assessment could be allocated to receive allo-HSCT in CR1 if a high level of MRD is detected after the induction therapy [29].

In conclusion, we observed that allo-HSCT offers significant OS and DFS benefits for higher-risk pediatric AML in CR1. Our findings support the indication for allo-HSCT in subgroups of newly diagnosed AML considered at greater risk of relapse, providing evidence to guide future protocol design. Enrollment in therapeutic trials must be encouraged to further corroborate the results of the present meta-analysis. In particular, randomized clinical trials comparing transplantation with other types of post-remission therapy are warranted. Moreover, it remains the need to further individualize allo-HSCT indications based on refined genetic, genomic, and MRD monitoring informed stratification.

## Supplementary Information

Below is the link to the electronic supplementary material.

Supplementary file1 (DOCX 114 KB)
