# Supplementary material for: Allogeneic hematopoietic stem cell transplantation for pediatric acute myeloid leukemia in first complete remission: a meta-analysis
Source: Ann Hematol. 2022 Aug 30;101(11):2497–506. doi: 10.1007/s00277-022-04965-x (PMC9546991; doi:10.1007/s00277-022-04965-x)
Supplement: Supplementary file 1 — Supplementary file1 (DOCX 114 KB) [file 277_2022_4965_MOESM1_ESM.docx]

| Study Group | Study Protocol | Reference | Risk stratification | Down Syndrome Patients | allo-HSCT indication | Chemotherapy group consolidation treatment | Conditioning regimen | Time of follow up (years) |
| --- | --- | --- | --- | --- | --- | --- | --- | --- |
| Japan | AML 99 | Tsukimoto et al. 2009 | HR: no CR after consolidation course 1 or induction C or with abnormalities of monosomy 7,5q-, t(16;21), t(9;22).  IR: not in either a low-risk or high-risk group.  LR: t(8;21) and WBC lower than 50,000, inv(16), or an age younger than 2 years without high-risk factors. | No | HR from any donor  IR if a MFD was available | Randomly assigned between four courses of consolidation chemotherapy plus auto HSCT versus five courses of chemotherapy. Auto-HSCT arm stopped in June 2002 | not reported | 5 |
| SJCRH | AML 02 | Rubnitz et al.. 2010 | HR: >25% blasts after induction 1 or persistent MRD after three courses of therapy.  SR: All other patients  LR: t(8;21)/*AML1-ETO*, inv(16)/*CBF*β*-MHY11*, or t(9;11)/*MLL-AF9* | No | HR from any donor  IR if a MFD was available  Comparison only in HR | Three courses of cytarabine-based chemotherapy | not reported | 3 |
| BFM | AML 1998 | Klusman et al. 2012 | HR: Patients with FAB M1/2, t(8;21), M4eo or inv(16) and more than 5% blasts in the bone marrow at day 15 (centrally reviewed) and patients with all other subtypes.  SR: all patients with AML FAB M1/2 and Auer rods, M3, M4eo, t(15;17), t(8;21) or inv(16). | No | If a MFD was available  Comparison only in HR and including both MFD and MUD | One course of HAE (high-dose cytarabine and etoposide) as intensification therapy and maintenance therapy for 12 months (thioguanine, cytarabine, intrathecal cytarabine). | Bu/CY | 5 |
| AIEOP | AML 2002/01 | Pession et al. 2013 | HR: non standard risk patients  SR: isolated anomalies of CBF and CR after first induction course. | No | HR if a MFD was available. If< 1 year of age, with AML-M7, or not in CR at the end of first ICE, or FLT3- ITD or with a complex karyotype from any donor | Auto HSCT with in vitro marrow purging with mafosfamide | Bu/CY/L-PAM | 8 |
| COG | POG 9421,  CCG 2961,  AAML03P1 | Kelly et al. 2014 | HR: monosomy 7/del7q, monosomy 5/del 5q, abnormalities of 3q, t(6;9)(p23;q34), or complex karyotype)  LR: t(8;21)(q22q22), inv(16)(p13;q22), and t(15;17) (q22;q21) | No | HR from any donor | Consolidation chemotherapy depending on each separate trial | POG 9421: TBI/etoposide  CCG 2961: Bu/CY  AAML03P1: not reported | 5 |
| COG | AAML0531 | Gamis et al. 2014 | HR: FLT-3 internal tandem duplication high allelic ratio, monosomy 7, monosomy 5/5q deletion, or persistent disease (PD) at the end of IND1)  IR: absence of low- or high-risk factors  LR: t(8;21)(q22;q22), inv(16)(p13.1q22), or t(16;16)(p13.1;q22) | No | HR from any donor  IR if a MFD was available. Comparison only in IR. | Two cycles of intensification chemotherapy | Bu/CY | 3 |
| Japan | AML 05 | Hyakuna et al.2019 | HR: either monosomy 7, 5q−, t(16;21), Ph+, FLT3-ITD, or induction failure after the first course of chemotherapy)  IR: all other patients  LR: core-binding factor AML without *FLT3*-ITD and good response to the initial induction chemotherapy | No | HR from any donor | Two more cycles of consolidation chemotherapy | Bu/L-PAM 35%  TBI/CY 41 %  Others 24% (6% RIC) | 3 |
| Uruguay | LAM 08 | Dufort y Alvarez et al. 2020 | HR: complex karyotype ( ≥ 3 clonal cytogenetic alterations), monosomy 7, monosomy 5/del (5q), FLT3-ITD, or mega- karyoblastic leukemia and nonresponders to 2 induction treatments.  IR: all the other patients  LR: t(8;21)/RUNX1-RUNX1T1, inv (16)/CBFβ-MHY11, or t(16;16)/CBFβ-MHY11 and complete remission (CR) after 1 or 2 induction treatments | No | HR from any donor  IR if a MFD was available. Comparison only in IR. | Two more consolidations with high doses of Ara-C and etoposide | Bu/Fludarabine/L-PAM or Bu/CY/L-PAM or RIC with Fludarabine/Thiotepa/L-PAM | 5 |
| South Korea | Different protocols | Lee et al. 2021 | Cytogenetics according to BFM. | 4,9% percent, none of them received HSCT | Performed depending on the associated risk, donor availability, and institutional policy.  Comparison only in HR and IR. | Maintenance chemotherapy in some cases | not reported | 5 |

Supplementary Table 1: Additional description of included studies

*Bu= Busulfan; CY= Cyclophosphamide, L-PAM= Melphalan, RIC= Reduced Intensity Conditioning, TBI= Total Body Irradiation*

Search String:

(Bone marrow transplant* OR BMT OR stem cell transplant* OR SCT OR hematopoietic transplant* OR haematopoietic transplant* OR hematopoietic stem cell transplant* OR haematopoietic stem cell transplant* OR HSCT) AND (acute myeloid leukemia OR acute myelogenous leukemia OR AML OR acute myeloid leukaemia OR acute myelogenous leukaemia) AND (children OR childhood OR pediatric) AND (complete remission OR CR OR first complete remission OR 1 CR OR first line OR newly diagnosed)
